# Chrononutrition in Cardiometabolic Health

**DOI:** 10.3390/jcm11020296

**Published:** 2022-01-07

**Authors:** Vasiliki Katsi, Ilias P. Papakonstantinou, Stergios Soulaidopoulos, Niki Katsiki, Konstantinos Tsioufis

**Affiliations:** 1 Cardiology Department, School of Medicine, Hippokration General Hospital, National and Kapodistrian University of Athens, 157 72 Athens, Greece; vkkatsi@yahoo.gr (V.K.); ktsioufis@gmail.com (K.T.); 2 Internal Medicine Department, Evangelismos Hospital, 106 76 Athens, Greece; iliaspapacon@yahoo.gr; 3 First Department of Internal Medicine, Medical School, AHEPA Hospital, Aristotle University of Thessaloniki, 541 24 Thessaloniki, Greece; nikikatsiki@hotmail.com

**Keywords:** cardiovascular disease, chrononutrition, circadian rhythms, intermittent fasting, time-restricted eating

## Abstract

In recent years, a healthy balanced diet together with weight reduction has risen to the forefront of minimizing the impact of cardiovascular disease. There is evidence that metabolic processes present circadian rhythmicity. Moreover, the timing of food consumption exerts a powerful influence on circadian rhythms. In this context, the subject of chrononutrition, described as the alignment of timing of food intake to the rhythms imposed by the circadian clock, has attracted considerable interest for possible beneficial effects on cardiovascular health. Current human studies suggest that chrononutrition-based dietary interventions could reduce the risk for cardiovascular disease by improving weight control, hypertension, dyslipidemia, and diabetes. However, meta-analysis of randomized control trials in this topic present varying and somehow conflicting results. Even the traditional association of breakfast skipping with adverse cardiovascular outcomes is nowadays controversial. Therefore, long-term and fairly consistent studies on the effect of chrononutrition on cardiovascular outcomes are needed. The purpose of this review is to provide concise evidence of the most recent literature involving the effects of chrononutrition and the specific chrononutrition-based dietary interventions, in particular time-restricted eating, on body weight and other cardiovascular disease risk factors.

## 1. Introduction

The prevailing risk factors for cardiovascular disease (CVD), namely obesity, diabetes mellitus, and hypertension, along with the associated unhealthy poor-quality diet imposed by lifestyle constraints, if effectively optimized, would markedly reduce the prevalence of CVD complications and the subsequent mortality [1,2]. Therefore, a healthy balanced diet together with weight reduction, in recent years, has become the cornerstone of minimizing the impact of CVD [3]. People nowadays understand the importance of adopting health-related behaviors and overcoming previously unhealthy diet prototypes and lack of physical activity [4]. Interestingly, smartphone-based methods and wearable devices are more and more used to measure physical activity and energy balance in real living conditions, with high levels of success in body weight reduction and improvement in overall quality of life [5,6].

Cardiometabolic health is dependent not only on the composition of a macromolecular diet, but even more importantly, on the distribution of energy, the frequency and regularity of meals, and the duration of the eating period and fasting within the day [7]. There is evidence that the metabolic processes present circadian rhythmicity, and the timing of food consumption exerts a powerful influence on circadian rhythms [8]. This is important because, as suggested by emerging research, irregular food timing may increase energy intake and reduce energy expenditure [7], thus promoting the development of obesity and other metabolic disorders [9,10]. Characteristic paradigms of irregular eating habits, such as breakfast skipping and late eating, are associated with increased risk of incident of heart disease and obesity [11].

In this context, ‘chrononutrition’ as a term synonymous to improving metabolic health, has recently attracted considerable interest. Chrononutrition represents a principle that describes the alignment of timing of food intake to the rhythms imposed by the circadian clock, in favor of metabolic processes involved in nutrition [7,12,13]. From this perspective, the term chrononutrition could be further extended to include timed eating dietary interventions, aiming at cardiometabolic benefit by the adjustment of food intake to the circadian rhythm [7,14].

Current human studies suggest that chrononutrition interventions present varied and conflicting effects on cardiovascular outcomes, and further studies are needed to draw safe conclusions. The purpose of this article is to explore the anticipated benefit in cardiometabolic health of chrononutrition. Therefore, we synthesized a brief review of the most recent evidence involving the effects of chrononutrition and the specific chrononutrition-based dietary interventions on body weight and other cardiovascular disease risk factors. The array of these chrono-nutritional interventions includes intermittent fasting (IF) and the time-restricted eating (TRE), a distinct form of IF [15,16]. The focus of this review will be TRE, over the forms of IF, because TRE involves an element of restricted food timing within the day, optimally aligned to the biological rhythmicity imposed by the circadian clock. 

## 2. Chrono-Nutritional Interventions and Their Novelty

Among the chrono-nutritional interventions, IF encompasses a period of fasting and a period of normal unrestricted eating (ad libitum eating) during the same week; there are two forms: (i) alternate day fasting (ADF), with calorie intake ranging between zero (complete fasting) and very low, up to 40%, on fast days [17]; and (ii) complete abstinence from calories or severe restriction on fasting days, such as ~500 kcal on 2 consecutive days per week, named the 5:2 diet [16]. TRE, in general, proposes restraining the feeding window from 6 to 10 h and fasting for the remaining hours per day, to match it with the circadian clock [18].

Chrononutrition-related interventions present beneficial effects on metabolism, weight loss, blood pressure, and cardiovascular health [18,19,20,21,22,23]. A direct comparison regarding the two forms of intermittent fasting and the time-restricted eating and their reported potential cardiovascular benefits can be found on Table 1. These aspects highlight chrononutrition as a meaningful novel modification for individually-tailored weight management programs that enable adaptability to lower-intensity long-term lifestyle changes for weight reduction. Furthermore, this approach is important for another reason; it may confer cardiovascular benefit without drugs used for weight management [23]. This is due to difficult long-term adherence, safety concerns, and disappointing results for sustaining weight after drug discontinuation [24]. In addition, no anti-obesity drugs have demonstrated a reduction in major adverse cardiovascular events or outcomes [25].

## 3. The Effects of Food Timing in Metabolism

Evidence from both animal and human studies indicates that the timing of food intake during the day can impact metabolic rhythms that are vital for human health [13]. Food intake displays a profound effect, through input signals on molecular oscillators, called the circadian clocks, which are found in almost every cell and tissue and entrain a rhythmic coordination of the metabolic processes [26]. It is nowadays well described that eating at “irregular” time can result in a desynchrony of the circadian system [18,27]. This circadian misalignment, due to mistimed eating, results in abnormal metabolic regulation/homeostasis, and consequently, increased cardiometabolic risks, including the development of obesity, type 2 diabetes, and ultimately CVD [28,29]. The exact mechanisms are likely to involve the transmission of multiple reinforcing signals, the expression of many energy-regulating endocrine hormones, and also alterations in adipose tissue regulation, all of which contribute to promoting obesity [27,30]. Notably, metabolic hormones that communicate meal timing to circadian clocks, and therefore regulate energy metabolism, include cortisol, insulin, insulin-like growth factor 1 (IGF-1), ghrelin, leptin, pro-opiomelanocortin (PYY), gastric inhibitory polypeptide (GLP-1), and adiponectin [27]. These hormones demonstrate circadian rhythmicity; their peak circulating levels present time-of-day-dependent variation, essential for more efficient nutrients metabolism [18]. This action appears optimal earlier in the day rather than during rest hours [18]. Characteristically, cortisol attains a peak at 8 a.m.; ghrelin, which increases appetite, at 8 a.m., 1 p.m., and 6 p.m.; adiponectin at 11 a.m.; insulin at 5 p.m.; and leptin, which inhibits fat accumulation, at 7 p.m. Under this scope, we believe, TRE, if applied early in the day, could be better aligned to the pulsatile rhythm of circadian clock and be beneficial. Among the energy-regulating hormones, the role of adiponectin is of particular importance and is discussed in TRE studies, as will be further mentioned. Adiponectin is secreted by adipocytes, exhibiting anti-diabetic, anti-inflammatory, and anti-atherogenic effects, while acting as an insulin sensitizer [31]. The reduction of adiponectin levels displays a central role in obesity, insulin resistance, progression of type 2 diabetes, hypertension, and CVD, while weight loss or caloric restriction leads to increasing adiponectin levels, and this increase is associated with increased insulin sensitivity [32].

## 4. Abnormal Eating Patterns and Health Outcomes

Meal irregularity or abnormal eating patterns are defined as more frequent eating events over a longer period of time throughout the day and at different times from 1 day to the other [33]. From meal timing studies, it is estimated that 50% of adults have a daily eating window that exceeds 14 h with no distinction in the traditional/natural breakfast-lunch-dinner pattern [5]. Moreover, irregular meal timing is followed by greater distribution of daily energy intake to the evening, increased frequency of eating occasions, and extended duration of daily eating periods [33,34]. A characteristic paradigm of irregular eating patterns is breakfast skipping. Regular breakfast consumption is associated with improved nutritional status throughout the rest of the day, including the lowest added sugar intakes [35]. Evidence derived from epidemiological studies suggests that skipping breakfast is associated with increased risk of heart disease [36], type 2 diabetes [37], obesity [38], and mortality from CVD [39]. However, despite these associations, current systematic reviews and meta-analyses of randomized controlled trials (RCTs) that evaluated breakfast skipping compared with breakfast consumption, reported minimal evidence that breakfast skipping might lead to weight gain and the onset of overweight and obesity [40], or negatively affect other cardiometabolic risk factors [41]. Moreover, a recent meta-analysis that evaluated breakfast consumption, body weight, and energy intake, supported that participants assigned to breakfast had a higher total daily energy intake than those assigned to skip breakfast [42]. Given the controversy of these results, the association of breakfast skipping to the development of obesity and cardiovascular disease remains to be evaluated in further studies.

## 5. Impact of Time Restricted Eating in Cardiometabolic Parameters

The chrononutrition-based intervention of TRE represents a meaningful novel modification for weight management in obesity, but also exhibits pleiotropic metabolic benefits through an intrinsic effect on circadian rhythms. It is therefore considered promising for the improvement of several key indicators of CVD [43,44,45,46]. The current literature involving RCTs and meta-analysis, examining the relationship between TRE and CVD outcomes in humans, is limited, and there are only a few RCTs with low risk of bias [46]. Noteworthy, regarding important parameters and mainly gender and age that are directly related to the metabolism and CVD development, no significant differences were reported among the participants in the studies examined. Thereafter, in the next few paragraphs, we discuss the impact of TRE on weight control, hypertension, dyslipidemia and diabetes, and overall CVD. 

### 5.1. Time Restricted Eating and Effects in Adiposity and Obesity 

Adiposity and, in particular, visceral adipose tissue, is an independent risk factor for cardiovascular morbidity and mortality, type 2 diabetes, atherosclerosis, and cardiovascular disease [47]. The evidence from most human studies suggests that IF and TRE generally result in weight loss [46,48]. A meta-analysis of both randomized and non-randomized controlled trials that compared TRE to a regular diet with 294 participants following TRE showed a significant reduction in body weight [46]. Interestingly, this was evident in participants with a metabolic abnormality, while the subgroup with healthy participants did not show a significant change of body weight [46]. Individuals with obesity present mild reductions in weight loss (1–4%) after 1–16 weeks of 4–10 h/day TRE, and this weight loss results from unintentional reductions in energy intake (~350–500 kcal/day) [49]. In another study including obese adults, an 8 h/day TRE allowing unrestricted energy intake resulted in a decrease in the number of eating occasions by ~20%, suggesting that this may be the case of involuntary reductions of energy intake in TRE [50]. Interestingly, TRE can lead to weight loss even without deliberate calorie restriction [5,51,52].

On the contrary, other studies of TRE report no effects on body weight. An intervention with 8 h TRE for 12 weeks in overweight and obese individuals, compared with controls receiving isoenergetic continuous hypocaloric diets or consistent meal timing, showed no differences in weight loss, estimated energy intake, and other secondary outcomes between groups [53]. In a small sample size study of overweight pre-diabetic men where early (between 8 a.m. and 6 p.m.) TRE (eTRE) was implemented, there was an improvement in insulin sensitivity and β-cell responsiveness, although no effect on weight loss and glucose levels was observed [54]. Another study examined the effects of eTRE versus standard dietary advice and found no differences in weight loss and energy intake, while an improvement of glycemic control, independent of weight loss, was noted. According to other authors, these metabolic benefits of eTRE could represent chronic adaptations [55]. At this point, we note that TRE was found to be effective in improving glycemic control regardless of the time it was implemented during the day, in a study that examined this issue [56]. Finally, in a randomized controlled trial (RCT) that compared the metabolic benefits of 12 h TRE to standard dietary advice in adults with metabolic syndrome, there was no significant difference in weight loss between groups. The duration of eating in this study was extended, providing an explanation for the inefficiency of the 12 h TRE in weight loss [57]. In fact, data about changes in body weight according to the timing of the eating window are limited, due to the novelty of the topic and the difficulty of following TRE in daily life. Although, in general, 4–10 h TRE is considered to induce modest (4%) weight loss in overweight and obese subjects, it does not appear that the shorter eating windows (4–6 h) produce greater degrees of weight loss compared to longer eating windows (8–10 h) [49,58].

Concerning outcomes of TRE in body composition, data on the changes in fat mass and fat-free (lean) mass are also conflicting. Participants following TRE showed significantly reduced body weight and fat mass [46,59], and this effect was attributed to the increase in adiponectin levels. Other studies are unsupportive of drastically different fat loss by TRE from controls [5,52,58]. In a recent study assessing the effects of TRE on fat mass and visceral fat in overweight adults, after adjusting for body weight loss, no significant changes on these parameters were observed, indicating that TRE effect is mediated by body weight loss [50]. In this study, the TRE group had a significant reduction in lean (muscle) mass mainly from the legs, with no significant lean mass loss from the trunk or arms. The effect of TRE on lean mass loss remained non-significant even after adjusting for body weight loss [50]. Findings from other studies showed preserved fat-free mass [46] and that a 4–8 h TRE induces a spontaneous calorie restriction and significantly lowers fat mass without changing muscle mass in young resistance-trained adults [60].

### 5.2. Time Restricted Eating and Change in Glucose Metabolism—Diabetes

TRE has been shown to significantly lower fasting glucose levels, fasting insulin levels, and insulin resistance, while increasing insulin sensitivity, although this was not universally observed [15,61,62,63]. The beneficial effects regarding glycemic control appear more evident in early (between 8 a.m. and 2 p.m.) TRE (eTRE). Studies showed that eTRE improves whole-body insulin sensitivity, increases skeletal muscle glucose uptake, reduces 24-h glucose levels, and improves lipid metabolism, regardless of caloric restriction or weight loss [54,55,58,64,65,66]. This result reported to be due to a significant increase of adiponectin [67]. Recent available research suggests that overall the IF regimens that incorporated TRE were well tolerable as a non-medicinal treatment option for patients with type 2 diabetes, and patients were able to reverse their need for insulin therapy during therapeutic IF/TRE protocols [68]. Nonetheless, the beginning of an IF/TRE regimen in diabetic patients should be under supervision by their physician for titration of the patients medication and important safety advice, in order to avoid adverse effects [68,69].

### 5.3. Effects of Time Restricted Eating on Lipid Profile

As shown in large public health databases, starting energy consumption earlier in the day presents beneficial effects on lipid profile and cardiometabolic endpoints [70]. In most studies, participants following TRE showed beneficial effects on lipid profiles, suggesting a reduction in the CVD risk linked to dyslipidemia, but the observed results were highly variable [61]. However, in an analysis of 10 studies evaluating lipid profiles, there were no significant changes in LDL-C and HDL-C levels in participants following TRE, while triglycerides significantly decreased [46]. Based on available findings to date, TRE does not seem to modulate HDL cholesterol levels or inflammatory markers, such as CRP [49]. Furthermore, a study of 12 weeks of 8 h TRE from 10:00 to 18:00 h, with unrestricted (ad libitum) food intake, in comparison to a no-intervention control group, found that in obese patients the metabolic biomarkers LDL and HDL cholesterol, as well as triglycerides, were not significantly different compared to controls [52].

### 5.4. Effects of Time Restricted Eating on Blood Pressure 

Several studies suggest beneficial effects of TRE on blood pressure (BP) levels and report somewhat consistent BP reduction [46,49,52,54,59,71]. A significant reduction of systolic BP by 11 ± 4 and diastolic BP by 10 ± 4 mm Hg after a 6 h TRE dietary intervention for 5 weeks was noted in a small study in men with prediabetes [54]. In the same direction, a study using a smartphone application for capturing meal timing found a significant decrease in systolic BP (−12 ± 11 mmHg, *p* = 0.002) in addition to weight loss [72]. However, in a systematic review and meta-analysis investigating the effects of fasting and energy restriction on BP levels in a total population of 1400 participants, the average BP reduction of both systolic (−3.3 mm Hg) and diastolic (−1.6 mm Hg) BP was modest [73]. Decreases in BP were more commonly seen in studies where participants lost at least 3% of baseline body weight [52,71]. Other studies reported mixed findings on the effect of TRE on BP, relative to a non-TRE group [50,53,56,57,58], which is attributed maybe to numerous confounders, adherence to the intervention, length of TRE, and the method of BP measurement. 

## 6. Current Landscape on ‘Watch the Clock’ Diets 

The current literature on the influence of chrononutrition-based ‘Watch the clock’ diets with CVD events and outcomes is limited. We have compelling evidence for this association to draw meaningful and robust conclusions. Therefore, summarizing the pros and cons in this topic, we distinguished observations and the results from a meta-analysis of RCTs. Adafer et al. (2020) suggested in a meta-analysis from recent RCTs that TRE induced an average weight loss of 3% accompanied by a loss of fat mass, which was observed without any caloric restriction [74]. Furthermore, TRE produced beneficial metabolic effects independently of weight loss, or the nutrition quantity and quality, suggesting an intrinsic effect based on the realignment of feeding and the circadian clock [74,75]. However, only one study had a high level of evidence, with a controlled and randomized protocol and adequate sample size, while in the other trials the overall level of evidence was low to medium with short intervention periods and small samples [74]. In line with the results of this meta-analysis, another meta-analysis of 19 studies, including 11 RCTs with low risk of bias, demonstrated beneficials effects of TRE on cardiometabolic parameters [46].

Other authors question the scientific evidence regarding the long-term effects of IF/TRE in terms of safety, efficacy, and compliance due to a short intervention period with these regimens, and underline the inconclusive data concerning metabolic consequences. Lowe et al. stated that TRE, in the absence of other interventions, is not more effective in weight loss than eating throughout the day [53]. Finally, Allaf et al. (2021), in a meta-analysis of RCTs comparing different IF regimens to eating without time, caloric or continuous energy restriction, concluded that the chrononutrition-based regimens were superior to ad-libitum feeding in reducing weight, but this was not clinically significant, compared to continuous energy restriction in improving cardiometabolic risk factors [76]. Noteworthy, the problem with continuous energy-restriction diets is likely the observed increase in appetite and energy intake after an acute period of severe energy restriction to compensate the alteration in energy balance and to lessen the created energy deficit [77]. Furthermore, calorie restriction can lead to more weight lost in the form of fat-free mass (muscle mass), which subsequently decreases metabolic rate; therefore, calorie restriction diets require increased efforts to maintain weight loss over time [78].

## 7. Time Restricted Eating: Is It Safe?

TRE is arguably the “mildest” form, it is more feasible and easier to implement compared to the more intense IF regimens and the conventional dietary approach of daily caloric restriction; this could facilitate compliance [49]. In healthy non-obese midlife and older adults, TRE was safe and well-tolerated, with no adverse events in lean mass, bone density, or nutrient intake [66]. TRE of less than 10 h was also safely implemented in an older population with comorbidities assessed for cognitive impairment [79]. Thus, TRE appears to be safe and beneficial, even in older subjects or in subjects at risk for CVD; however, caution is advised for daily fasting periods lasting 14 h or longer [62]. 

## 8. Conclusions and Future Directions

The timing of food intake is an important factor for metabolic regulation in interaction with our circadian clock. Chrononutrition investigates the alignment of food intake with circadian rhythms, which exhibits, from preliminary findings, promising benefits on body weight reduction and other CVD risk factors. This may designate chrononutrition, particularly in the form of time-restricted eating, as an important component of a multidisciplinary approach to combat CVD. Nevertheless, we have compelling current evidence for the associations of chrononutrition with CVD risk factors. To draw meaningful and robust conclusions, future high-quality trials with solid design to detect significant differences in CVD outcomes, of longer duration (>6 months) and larger sample sizes, are expected to be conducted. It will be of interest for future research to examine how chrononutrion and TRE affect subjects with specific CVD risk factors. The applicability and benefits of ‘Watch the clock’ diets in the healthy population, aiming to reduce weight, and the benefit from possible anti-aging properties, should also be investigated. A comparison between TRE and other fasting regimens needs also to be further explored, as well as the sustainability of weight loss in the long term. The unraveling of these issues in the topic of chrononutrion may confer innovative accumulated evidence for the anticipated beneficial impact in CVD prevention.

## Figures and Tables

**Table 1:** Comparative data on potential benefits of intermittent fasting and time-restricted eating.

Dietary Intervention	Calorie Intake/ Weight Change	Glucose Metabolism	Lipid Metabolism	Other Effects	References
Time Restricted Eating (TRE) restriction of the daily eating window to 6–10 h, with 14–18 h of fasting per day	facilitated weight loss and appetite reduction in overweight and obese people	lowered insulin levels and produced better insulin sensitivity	decrease in LDL levels	BP decrease	[15,18,20]
Alternate-Day Fasting (ADF) calorie restriction only every other day 25% of the usual intake consumption on the fast day (approximately 500 kcal), alternated with ad libitum food consumption on the “feast day”	loss of 3.6–8.5% of body weight after 12 months	non-significant differences in fasting plasma glucose and insulin	non-significant improvements in lipid profiles	non-significant and modest BP reductions BP control needs further research	[17,20]
The 5:2 diet absolute fasting or severely restricting the caloric intake for 2 consecutive days per week with ad libitum consumption on the remaining 5.	reductions in body mass, fat mass, and fat-free mass	modest reductions in fasting insulin and insulin resistance	reductions in postprandial triglycerides concentrations	modest BP reductions BP control needs further research	[16]

BP: blood pressure; LDL: low-density lipoprotein.

## Data Availability

Not applicable.
